# Automatic disease prediction from human gut metagenomic data using boosting GraphSAGE

**DOI:** 10.1186/s12859-023-05251-x

**Published:** 2023-03-31

**Authors:** K. Syama, J. Angel Arul Jothi, Namita Khanna

**Affiliations:** 1 Department of Computer Science, Birla Institute of Technology and Science Pilani Dubai Campus, Dubai International Academic City , Dubai, UAE; 2 Department of Biotechnology, Birla Institute of Technology and Science Pilani Dubai Campus, Dubai International Academic City , Dubai, UAE

**Keywords:** Metagenomics, Disease prediction, Ensemble GNN, GraphSAGE, Machine learning, Deep learning

## Abstract

**Background:**

The human microbiome plays a critical role in maintaining human health. Due to the recent advances in high-throughput sequencing technologies, the microbiome profiles present in the human body have become publicly available. Hence, many works have been done to analyze human microbiome profiles. These works have identified that different microbiome profiles are present in healthy and sick individuals for different diseases. Recently, several computational methods have utilized the microbiome profiles to automatically diagnose and classify the host phenotype.

**Results:**

In this work, a novel deep learning framework based on boosting GraphSAGE is proposed for automatic prediction of diseases from metagenomic data. The proposed framework has two main components, (a). Metagenomic Disease graph (MD-graph) construction module, (b). Disease prediction Network (DP-Net) module. The graph construction module constructs a graph by considering each metagenomic sample as a node in the graph. The graph captures the relationship between the samples using a proximity measure. The DP-Net consists of a boosting GraphSAGE model which predicts the status of a sample as sick or healthy. The effectiveness of the proposed method is verified using real and synthetic datasets corresponding to diseases like inflammatory bowel disease and colorectal cancer. The proposed model achieved a highest AUC of 93%, Accuracy of 95%, F1-score of 95%, AUPRC of 95% for the real inflammatory bowel disease dataset and a best AUC of 90%, Accuracy of 91%, F1-score of 87% and AUPRC of 93% for the real colorectal cancer dataset.

**Conclusion:**

The proposed framework outperforms other machine learning and deep learning models in terms of classification accuracy, AUC, F1-score and AUPRC for both synthetic and real metagenomic data.

## Background

The human body is the habitat for trillions of diverse and complex microbes (microbiota or microbiome). These microbes reside in various body sites (skin, gut, ear, mouth, nose, stool etc.) and play a vital role in (a) shaping and controlling human health, (b) developing the human immune system and (c) affecting human metabolism [1]. The most abundant and the largely studied microbes are found in the human gut. Research works have found that the dysbiosis of the human gut microbiome can cause many host diseases like inflammatory bowel disease (IBD), colorectal cancer (CRC), obesity, diabetes, and liver cirrhosis [2]. Recently, due to the emergence of various next generation sequencing techniques, huge amounts of human microbiome sequence data are available. This in turn has become the motivation for developing computational methods to identify the relationships between bacterial composition and functions with the diseases.

The microbiome sequence data is used to characterize the microbiome profiles for a deeper analysis. Many tools such as QIIME [3], MetaPhlAn [4] are available for taxonomy profiling of the human microbiome sequence data. These tools provide the relative abundance of each taxonomic group or the operational taxonomic units (OTUs) present in the microbiome sequence data. Thus, a sample in the human microbiome metagenomic dataset is described by the abundance of the OTUs. This abundance of the different taxa of microbiomes are used as meaningful indicators for the disease status of each host sample [5].

Due to the availability of vast amounts of human metagenomic data, machine learning (ML) and deep learning (DL) techniques are being used to analyze and identify the relationships between different microbes and the relationship between microbes and host diseases. Recently, many works have been published in automatic disease prediction using human gut microbiome metagenomic data with ML and DL techniques [6–10]. These works essentially used ML algorithms like support vector machine (SVM), random forest (RF), deep forest (DF), extreme gradient boosting (XGB), multilayer perceptron (MLP), and DL methods like convolutional neural network (CNN) and autoencoders. The ML and DL methods have achieved relatively moderate results as they suffer due to high dimension of the metagenomic datasets. Thus, there is still room for improving the classification performance of these models.

Researchers have always tried to improve and optimize classification models to achieve better accuracy. Ensemble learning is a widely used technique to improve the classification accuracy [11]. It could also help with metagenomic datasets where a single classifier model might not obtain superior performance due to the huge dimension of the datasets. It aggregates two or more base classifiers to improve the predictive performance of the combined classifier. Thus, it overcomes the weakness of a single weak base classifier.

Though several ML and DL models are used for metagenomic disease prediction, it could be noted that the graph neural network (GNN) models such as Graph SAmple and aggreGatE (GraphSAGE) [12] and graph convolutional network (GCN) [13] have not been applied for this purpose. One possible reason might be that the metagenomic data lack the inherent graph structure, as GNNs require graph structured data as input. The previous works on metagenomic disease classification using ML and DL methods have used the abundance of the OTUs present in the human microbiome metagenomic sample as the features for the disease prediction problem. However, these works have failed to model the relationship between the samples in the dataset for disease classification purpose in the form of a graph.

Inspired by ensemble learning and GCNs, this work proposes a novel framework for automatic metagenomic disease prediction. The proposed framework involves three steps. Firstly, the distance matrix is calculated from the OTU table. Secondly, a metagenomic disease graph (MD-graph) is constructed using the distance matrix. The MD-graph models the samples as the nodes. The edges between the graph nodes are constructed based on the assumption that similar samples exhibit similar features. Finally, the Disease prediction Network (DP-Net) module is constructed with ensemble of GraphSAGE models. The DP-Net is used to predict the sample phenotype.

All things considered, the proposed framework put forward the following contributions.

- An ensemble model based on GraphSAGE for disease prediction using human gut microbiome metagenomic data.
- A novel graph construction method to construct the MD-graph from the gut microbiome metagenomic data.
- The efficiency of the proposed framework is studied by applying it to two different real disease datasets like IBD, and CRC. Also the model’s performance is assessed using synthetic datasets.

### Related work

This section aims to review the works done in disease prediction with human gut metagenomic data using ML and DL methods. The study done in [10] performed a comprehensive study by applying classical ML methods like SVM and RF for disease prediction to six metagenomic datasets spanning five diseases (type 2 diabetes (T2D), obesity, liver cirrhosis, CRC and IBD).

Subsequent to this, the work conducted in [14] analyzed various ML algorithms like SVM, RF, XGB, DF and an autoencoder pre-trained deep neural network (AutoNN) for disease prediction from metagenomic data. The work applied the ML and DL algorithms to six disease datasets to predict the sample status as healthy or sick. The authors used two features such as *k*-mer abundances and OTU abundances.

An automated software called MetaDP was developed by the study of [15] to perform data analysis of amplicon-sequenced metagenomic data for disease classification. The disease addressed in this work was intestinal bowel syndrome (IBS), and SVM was used as the classifier. In a similar work in [16], an ML framework called MicroPheno was proposed to classify host phenotypes as healthy or disease using metagenomic data. The Micropheno framework predicted Crohn’s disease using SVM and RF classifiers. In [17], SVM and MLP were used for classifying a given metagenomic sample into IBD or healthy class. The work combined two types of features such as the OTU abundances and gene group abundance and finally developed a hybrid classifier.

In [18], a DL model called metaNN was proposed to classify the host phenotype into IBD or healthy with an IBD dataset. This work used two neural networks (NN) models like MLP and a CNN, along with sample augmentation using statistical methods and microbe abundance profiles. The authors observed that data augmentation improved the classification performance of both ML and DL models. Also, results indicated that DL models are better than ML models as they perform automatic feature engineering.

In [19] a new method called met2Img was proposed to represent metagenomic data as images. The authors used T-distributed Stochastic Neighbor (t-SNE) embeddings for generating synthetic images from abundance data. Then, a CNN was used to classify five diseases. By converting each sample feature as images, the CNN could efficiently retrieve the patterns present in the sample. In another work by the same authors [20], a CNN was used for classifying samples from a CRC dataset from different cohorts such as Chinese, Austrian, American, German, and French into sick or healthy samples. A NN model called TaxoNN was proposed in [21], which utilized an ensemble of CNNs for disease prediction. TaxoNN applied the model on two disease datasets such as T2D and cirrhosis. The model incorporated a stratified approach to group the entire OTUs into phylum clusters, and different CNNs were trained within each cluster. Finally, the features obtained from each cluster were concatenated, which improved the classification accuracy of TaxoNN.

A CNN based model proposed in [9] implemented a new layer called phylo-conv layer to discriminate the subclasses of IBD. Patristic distance was used to find neighbors of taxa from the phylogenetic tree. Then, the phylogenetic tree OTUs were embedded in a euclidean space using multi-dimensional scaling. CNN was used to apply convolution over *k* nearest neighbors of the OTUs in the dataset. The proposed CNN reported promising results when compared with state-of-the-art ML algorithms. Another recent study by [8] proposed a model called pophy-cnn, which was built using a CNN to predict the host phenotype of sample from four different disease datasets. The biological information of taxa present in a sample is obtained from the phylogenetic tree. This information and the relative abundance value were embedded in a 2-dimensional matrix. Then, a 2-dimensional CNN was used for classification. The authors claimed that their model got significant improvement in performance when applied on nine disease datasets.

Table 1 presents a summary of the review done on the disease classification in metagenomic data. According to the literature, many works have used both ML and DL methods. Most of the works used CNN as the DL method for classifying metagenomic samples. There are few works which used GCN and variational auto encoders (VAE) for feature extraction and reduction from genomic data [22–25]. However, to the best of our knowledge, we observed that GNNs, such as GraphSAGE, have not been widely applied in the area of microbiome analysis to predict diseases. Moreover, none of the existing works have converted the OTU data into a graph and utilized the success rate of GraphSAGE in node classification.

In this work, the metagenomic data is converted to a graph called MD-graph using a novel graph construction method and a boosting GraphSAGE is used to predict the class label of samples in the dataset.

**Table 1:** Summary of work done on disease classification in metagenomics data using ML/DL methods

Reference	ML/DL approach(es) used	Diseases considered	Input features	Remarks/observations
[10]	SVM, RF	T2D, CRC, cirrhosis, IBD, obesity	OTU abundance	RF with feature selection outperformed basic RF and SVM classifiers with best AUC of 74%, 88.1%, 94.6%, 89.3%, 65.6% with T2D, CRC, cirrhosis, IBD, and obesity datasets respectively.
[14]	SVM, RF, XGB, DF, AutoNN	T2D, CRC, cirrhosis, IBD, obesity	OTU abundance, *k*-mer frequency	The proposed AutoNN model achieved the best accuracy of 66.3% using OTU feature on T2D dataset.
[15]	SVM	IBS	OTU abundance	The software package called metaDP can be used for classifying other disease samples.
[16]	SVM, RF	Crohn’s disease	*k*-mer frequency	The best F1-score of 76% was achieved by RF classifier with *k*-mer feature.
[17]	SVM, MLP	IBD	OTU abundance gene group abundance	The proposed hybrid classifier achieved an AUC of 80%
[18]	MLP, CNN	IBD	OTU abundance	The model achieved the best AUC of 89% with MLP by using data augmentation technique.
[19]	CNN	T2D, IBD, cirrhosis, CRC, obesity	OTU abundance	The model achieved the best accuracy of 84.2% and 66.3% for IBD and obesity datasets respectively.
[20]	CNN	CRC	OTU abundance	The model achieved the best AUC of 75.7% with CNN .
[21]	CNN	T2D, cirrhosis.	OTU abundance	The ensemble CNN achieved 76.2% AUC on T2D dataset and 91.1% on cirrhosis dataset
[9]	CNN	IBD	OTU abundance	The model ph-CNN achieved the best Matthews Correlation Coefficient (MCC) of 92%
[8]	CNN	IBD, T2D, obesity, cirrhosis	OTU abundance, phylogenetic relationship	The model PopPhy-CNN achieved the best F1-score of 58.7% for Obesity dataset.

## Methods

**Fig. 1:**
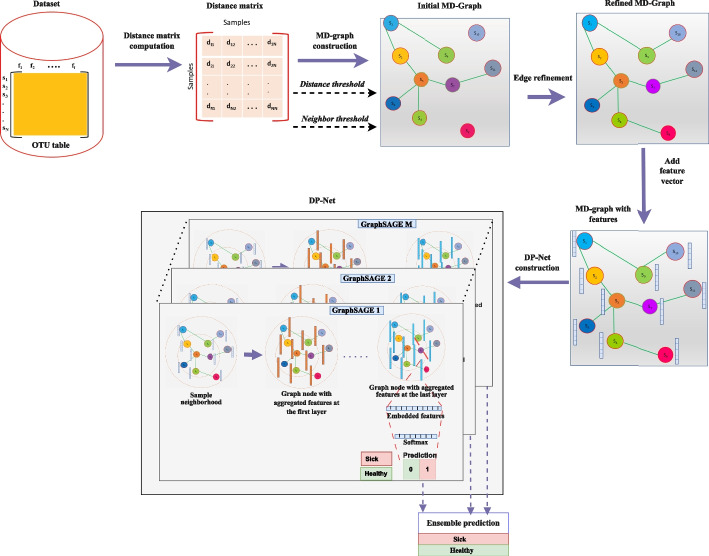
Architecture of the proposed framework

Disease prediction from metagenomic samples is the task of predicting if a given sample is healthy or sick based on the microbiome profile. The architecture of the proposed disease prediction framework is illustrated in Fig. 1. Given metagenomic samples, the aim of this framework is to learn the mapping between the human gut metagenomic samples and their labels using a boosting GraphSAGE and later use this knowledge to predict if a given metagenomic sample is healthy or sick. The framework consists of three stages, such as computation of the distance matrix, construction of the MD-graph and the label prediction using the DP-Net. The following sections define the problem and explain each stage of the proposed framework.

### The input dataset

The dataset in the form of an OTU table, along with the class labels, is given as input to the framework. For each sample in the dataset, the OTU table captures the species level relative abundance profiles (OTUs). The OTUs are considered as the features of a sample. Let *N* be the total number of samples in a dataset. Then, the set of samples in a dataset is denoted as $\{s_1, s_2, \dots , s_N\}$. A sample $s_i$ in a dataset be denoted as $s_i = (\varvec{f_i},y_i)$ where ${\varvec{f}}_i$ is the feature vector of $s_i$ and $y_i$ is the class label of $s_i$. The feature vector ${\varvec{f}}_i$ of a sample $s_i$ is of length *f* (i.e.) each sample contains a *f*-dimensional feature vector. Let the feature vector ${\varvec{f}}_i$ of a sample $s_i$ be $[ f_{i1},f_{i2},\dots , f_{if} ]$. Thus, the datasets are of dimension $N \times f$ matrix. In this work, the proposed framework is evaluated using both real and synthetic datasets.

#### Real datasets

Human gut metagenomic datasets of two different diseases, such as IBD [26] and CRC [27] are used. The IBD dataset contains 1359 samples and 1025 features. Out of the total samples, 336 are sick and 1023 are healthy. The CRC dataset contains 229 sick and 261 healthy samples with 319 features. The IBD dataset is affected by class imbalance. These are publicly available real datasets and are obtained using amplicon sequencing technique and contain the species level relative abundance.

#### Synthetic datasets

This work uses the technique implemented by [18] for generating synthetic data. It has been observed that the best fit to the OTU table obtained from amplicon sequence data is the negative binomial (NB) distribution [28]. Hence, in order to generate a synthetic dataset, a NB distribution is fitted for each class in the real dataset. The generated synthetic IBD data also follow the data imbalance in the real data. A detailed description of the datasets is given in Table 2.

**Table 2:** Description of the datasets used

Disease	Number of sick samples	Number of healthy samples	Number of features	Reference
*Real dataset*				
IBD	336	1023	1025	[26]
CRC	229	261	319	[27]
*Synthetic dataset*				
IBD_synthetic	3024	9207	1025	
CRC_synthetic	1145	1305	319	

### Distance matrix computation

The next step in the proposed framework is the construction of the distance (dissimilarity) matrix. The distance matrix *D* is a $N \times N$ matrix that captures the dissimilarity between the samples of the OTU table. The dissimilarity between the samples is based upon the feature values of the samples. Higher the dissimilarity between the samples, higher the dissimilarity value between them in the dissimilarity matrix and vice versa. An entry $D_{ij}$ in the distance matrix corresponds to the distance between samples $s_i$ and $s_j$ in the OTU table. Since the distance between a sample and itself is 0, the diagonal elements of the distance matrix are 0. A row of the distance matrix ${\varvec{d}}_i$ is a distance vector of sample $s_{i}$ and is of length *N* (i.e.) ${\varvec{d}}_i = [d_{i1}, d_{i2}, \dots , d_{iN}]$. Each element of the vector represents the distance of the sample $s_i$ to every other sample $s_j$ in the OTU table where $j = 1, 2, \dots , N$. In this work, the Cosine dissimilarity and the Manhattan dissimilarity metrics were used to compute the distance between the samples of the IBD and CRC datasets respectively. The choice of the dissimilarity metric used in this work for the datasets is found experimentally.

### Distance threshold computation

A distance threshold *t* is used in this work to determine if two graph nodes are neighbors. The distance threshold defines the maximum value for the distance between two graph nodes to become neighbors (i.e.) if the distance between two MD-graph nodes is less than or equal to the threshold *t* then, they are neighbors otherwise they are not neighbors. In this work, to begin with, we use the median of the values (*m*) of the distance matrix as the threshold value *t*. The reason behind selecting the median value as the threshold is that the median of a set of values mark the middle value. Moreover, choosing the median value resulted in inclusion of reasonable amount of neighbors (that is less than the maximum number of neighbors for a graph node) for all the graph nodes for all the datasets. If we do not use a distance threshold, then every graph node is considered as the neighbor of every other graph node. In that case, the resulting MD-graph would be too dense. The graph may also capture unnecessary relationships between nodes.

### Neighbor threshold computation

The neighbor threshold $\tau$ denotes the maximum number of neighbors for each MD-graph node. It is used in this work in order to eliminate the problem of standalone graph nodes and graph nodes with high degree. Standalone graph nodes are those graph nodes that do not have any neighbors. This situation may arise when a graph node’s distance with all its neighbors is greater than the distance threshold. Graph nodes with high degree are those graph nodes having too many neighbors. This situation may arise when a graph node’s distance with all its neighbors is lesser than the distance threshold. For a given metagenomic disease dataset, the maximum number of neighbors of each MD-graph node $\tau$ is fixed as some percentage of the total number of samples in a dataset (Perc$\_$val) and is given by Eq. 1. Hence, this value will vary from dataset to dataset. The optimal value of $\tau$ for each dataset is determined experimentally by varying the Perc$\_$val parameter.

1 $$\begin{aligned} \begin{aligned} \tau =\frac{Perc\_val * No. \,of \,samples \,in \,the \,dataset}{100} \end{aligned} \end{aligned}$$

While setting the value for $\tau$ for a dataset, the data imbalance problem is also considered. Imbalanced datasets are those datasets where the frequency of some class labels in the dataset is very less. For an imbalanced dataset, two neighbor thresholds $\tau _{sick}$ and $\tau _{healthy}$ are used. For a balanced dataset, a single neighbor threshold $\tau$ is used. This is necessary to avoid unwanted edges between a sick node and a healthy node. Let $\tau _{sick}$ denote the number of neighbors of a sick node and is fixed as some percentage of the total number of sick samples in the imbalanced dataset (Perc$\_$val) and is given by Eq. 2. Let $\tau _{healthy}$ denote the number of neighbors of a healthy node and is fixed as some percentage of the total number of healthy samples in the imbalanced dataset (Perc$\_$val) and is given by Eq. 3. The optimal value of $\tau _{sick}$ and $\tau _{healthy}$ for the imbalanced datasets is also determined experimentally. Since, the IBD dataset exhibit class imbalance problem, in this work, we use two separate neighbor thresholds for this dataset.

2 $$\begin{aligned} \tau _{sick} =\frac{Perc\_val * No. \,of \,sick \,samples \,in \,the \,dataset}{100} \end{aligned}$$

3 $$\begin{aligned} \tau _{healthy}=\frac{Perc\_val * No. \,of \,healthy \,samples \,in \,the \,dataset}{100} \end{aligned}$$

### Metagenomic disease graph (MD-graph) construction

After obtaining the distance matrix, the next step is to construct the MD-graph for a dataset. This is a challenging task because unlike the previous work where the GNNs have been applied on datasets with inherent graph structure, this work aims to apply GraphSAGE to metagenomic disease datasets that lack graph structure. The graph structure is imposed to the raw OTU table as follows: The samples in a dataset $\{s_1, s_2, \dots , s_N\}$ are assumed to be the nodes $\{v_1, v_2, \dots , v_N\}$ of the MD-graph *G*. The distance matrix *D* is used to construct the edges between the MD-graph nodes. The assumption is that similar graph nodes exhibit similar features and hence will belong to the same class (healthy/sick). Hence, in the MD-graph *G* an edge is present between similar graph nodes $v_i$ and $v_j$. Once the dataset is modeled as a MD-graph, the adjacency matrix *A* is obtained from it. In this work, the MD-graph is an unweighted, undirected graph with *N* nodes. The steps in the MD-Graph construction are detailed in the following subsections.

#### Edge construction

The distance matrix *D*, the neighbor threshold $\tau$, $\tau _{sick}$ and $\tau _{healthy}$, the distance threshold *t* and an empty adjacency matrix A of size $N \times N$ are given as the input to the MD-graph construction algorithm. The algorithm inserts edges between a graph node $v_i$ and its neighbors as follows:

Let $\tau _i$ denote the current maximum number of neighbors of a graph node $v_i$ and is initially set to 0. The initial value of *t* is set to $'m'$, where m is the median of values in the distance matrix of the corresponding dataset. The distance vector of the node $v_i$ is sorted. Let ${\varvec{d}}_i^{'}$ denote the sorted distance vector of graph node $v_i$ (i.e) ${\varvec{d}}_i^{'} = [ d_{i1}^{'}, d_{i2}^{'}, \dots , d_{iN}^{'}]$. Every element of the sorted distance vector is then compared with the distance threshold *t*. An edge is drawn between graph nodes $v_i$ and $v_j$ iff the distance between a graph node $v_i$ and its neighbor $v_j$ denoted as ${\varvec{d}}_{ij}^{'}$ is less than or equal to the distance threshold *t* and the current maximum number of neighbors of the node is less than or equal to $\tau$ and is given by Eq. 4.

4 $$\begin{aligned} A_{ij}= {\left\{ \begin{array}{ll} 1,&{} \text {if }\, (d_{ij}^{'} \le t) \cap (\tau _i \le \tau )\\ 0, &{} \text {otherwise} \end{array}\right. } \end{aligned}$$

The value of $\tau _i$ is incremented by 1 as soon as an edge is inserted between a graph node and its neighbor. This procedure is repeated for all the graph nodes.

The above procedure can be applied to graph nodes of imbalanced dataset. However the condition to insert an edge is given by Eqs. 5 and 6 for sick and healthy graph nodes respectively.

5 $$\begin{aligned} A_{ij}&= {\left\{ \begin{array}{ll} 1,&{} \text {if }\, (d_{ij}^{'} \le t) \cap (\tau _i \le \tau _{sick})\\ 0, &{} \text {otherwise} \end{array}\right. } \end{aligned}$$

6 $$\begin{aligned} A_{ij}&= {\left\{ \begin{array}{ll} 1,&{} \text {if }\, (d_{ij}^{'} \le t) \cap (\tau _i \le \tau _{healthy})\\ 0, &{} \text {otherwise} \end{array}\right. } \end{aligned}$$

Algorithm 1 shows the edge construction process for both balanced and imbalanced datasets.

#### Edge refinement

We observe that the above edge construction procedure at times result in a MD-graph where few graph nodes have less than the specified number of neighbors (sparse nodes). In some cases we also observed few standalone nodes in the MD-graph. The edge refinement step is used to handle standalone and sparse graph nodes.

Let *S* denote the set of all standalone and sparse graph nodes obtained after the edge construction process. In order to ensure that every node $v \in S$ has the specified number of neighbors, we gradually increase the threshold value *t* in steps and invoke the edge construction process on the graph node *v* until it acquires

- $\tau$ neighbors if the dataset is balanced (or)
- $\tau _{sick}$ neighbors if the dataset is imbalanced and *v* is a sick graph node (or)
- $\tau _{healthy}$ neighbors if the dataset is imbalanced and *v* is a healthy graph node

At every step, the *t* value is incremented by $\delta$ as given by Eq. 7.

7 $$\begin{aligned} t = t + \delta \end{aligned}$$

where, $\delta$ = $(max_{val} - m)/5$ where, $max_{val}$ is the maximum value of the distance matrix and *m* is the median of the distance matrix values.

#### Adding the feature vectors to MD-graph nodes

After the edge refinement step we obtain the MD-graph. For the purpose of GraphSAGE, the graph nodes are assumed to contain features. In this work, the OTU feature vector ${\varvec{f}}_i$ of a sample $s_i$ in the dataset is considered as the feature vector of the corresponding MD-graph node $v_i$. Similarly, the label $y_i$ of a sample $s_i$ in the dataset is considered the label of the MD-graph node $v_i$. The feature vectors of all graph nodes constitute the initial feature matrix $F^0 \in {\mathbb {R}} ^{N \times f}$ of the MD-graph.

The algorithm for the edge refinement and adding the feature vectors to MD-graph nodes is illustrated in Algorithm 2.
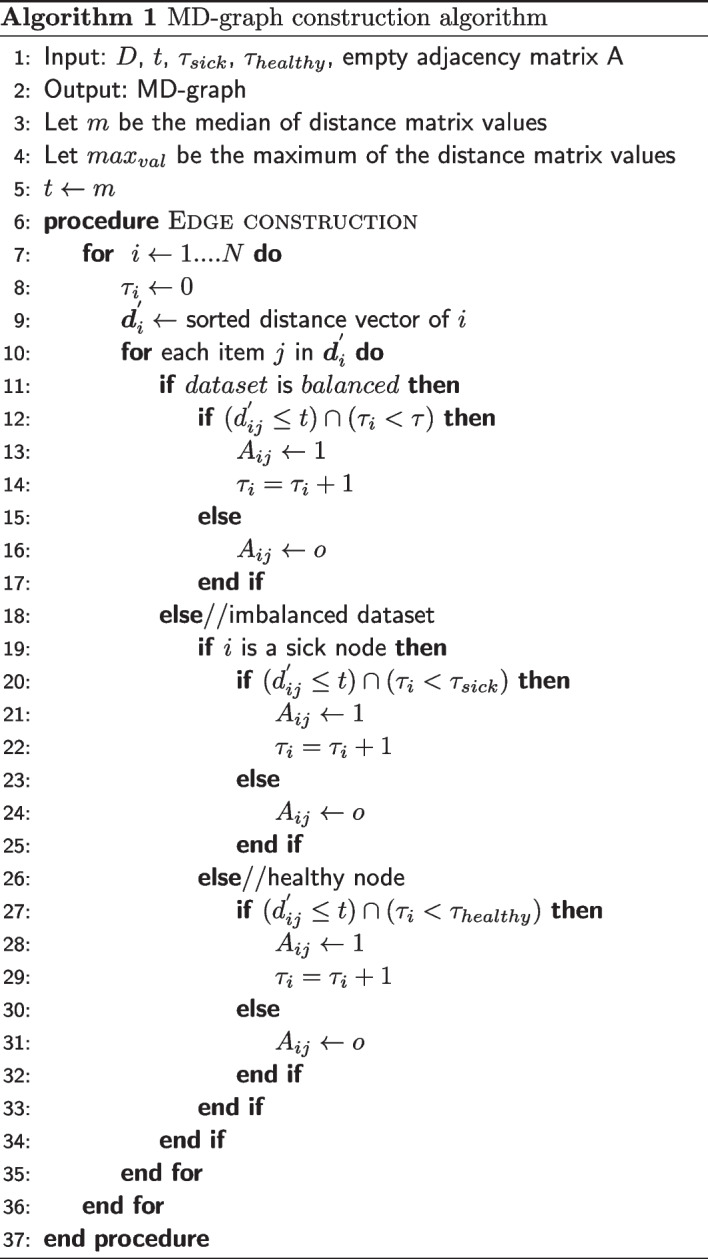

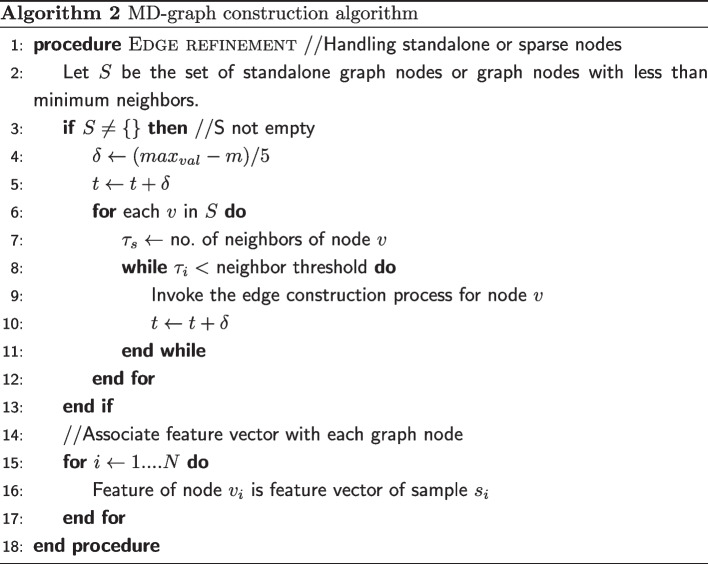


### Disease prediction network (DP-Net)

#### GraphSAGE

The GraphSAGE model utilizes the inherent structure of the graph structured data to generate feature representations of graph nodes. The node feature representations are helpful for prediction and graph analysis tasks. GraphSAGE uses an inductive approach, where the model discovers rules from the train samples, which are then applied to the test samples. Also, GraphSAGE has two improvements to the original GCN. Firstly, unlike the full graph training used in GCN, GraphSAGE uses a small batch training method by sampling the neighbors of a graph node. Secondly, the algorithm implements different aggregator functions for aggregating the features of the neighbors. Thus, GraphSAGE generates feature representation for each node by sampling and aggregating the features of nodes in its neighborhood. This is based on the idea that nodes in the same neighborhood should have similar features.

A GraphSAGE model is made up of several graph convolution layers. A graph convolution layer $k\in \left\{ 1,2,\cdot \cdot K\right\}$, where, *K* is the total number of graph convolution layers in the GraphSAGE model. Each graph convolution layer has a set of parameters like a sample number ($S_{k}$), a neighborhood value (*k*), a weight matrix ($W^{k}$), and a non-linear function ($\sigma$). The sample number denotes the number of neighbor nodes to be considered while generating node feature representations. The neighborhood value depends upon the number of the graph convolution layer. For the first graph convolution layer, the neighborhood value is 1. For the second graph convolution layer, the neighborhood value is 2, and so on. Finally, for the last graph convolution layer, the neighborhood value is K.

The GraphSAGE algorithm operates on a graph *G* where each node in *G* is associated with a feature vector ${\varvec{f}}$. It involves both forward and backward propagation. During forward propagation, the information relating to a node’s local neighborhood is collected and used to compute the node’s feature representation.

The forward propagation iterates over the search depth *K*. At each graph convolution layer $k\in \left\{ 1,2,\cdot \cdot K\right\}$ the following steps are done for all the graph nodes.

**Fig. 2:**
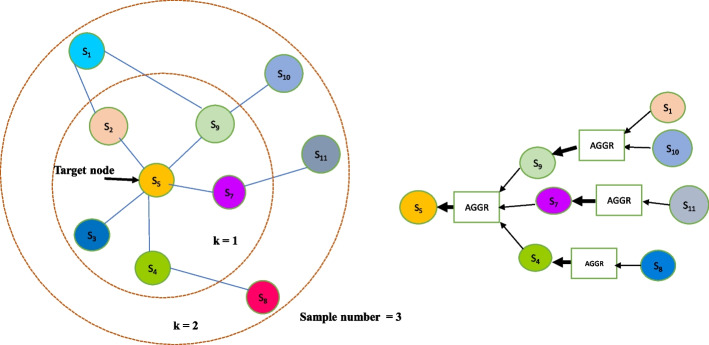
Architecture of the GraphSAGE model: The left side of the figure is a simple graph structured data and the right side of the figure shows the aggregation process of the target node. This architecture shows a two-layer GraphSAGE with a sample number of 3

- Choose a target node, $v\in V$, where *V* is the set of all nodes in the graph. Sample the neighborhood of the target node using the sample number and the neighborhood value of the graph convolution layer. This will result in a sampled neighborhood *N*(*v*). Then, aggregate the features of all the nodes in the sampled neighborhood using a differentiable aggregator function $AGGR_{k}$ to form the aggregated neighborhood representation $h^{k}_{N(v)}$, of node *v*. This is denoted by Eq. 8.
  8 $$\begin{aligned} h^{k}_{N(v)} = AGGR_{k} \left( \left\{ h^{k-1}_{u}, \forall u \in N(v)\right\} \right) \end{aligned}$$
  where *u* is one of the nodes from the sampled neighborhood of node *v* and $h^{k-1}_{u}$ represents the previous layer feature representation of node *u*. In this work, the Mean aggregator function is used to aggregate information from neighborhood nodes. The mean aggregator takes the elementwise mean of the vectors in a nodes sampled neighborhood. Figure 2 illustrates the sampling and the aggregation of node features by GraphSAGE model.
- Concatenate the previous layer representation of the target node $h^{k-1}_{v}$ with the aggregated neighborhood representation. Apply the linear transformation to the concatenated representation using a weight matrix $W^k$ associated with the layer. Pass the linear transformed representation to a nonlinear activation function $\sigma$ to obtain the new feature representation of the target node ($h^k_{v}$). The operations done at this step is given by Eq. 9.
  9 $$\begin{aligned} h^{k}_{v} = \sigma \left( W^{k} \cdot CONCAT \left( h^{k-1}_{v}, h^{k}_{N(v)}\right) \right) \end{aligned}$$
- Normalize the node representation to prevent gradient explosion. This is given by Eq. 10.
  10 $$\begin{aligned} h^{k}_{v} = \frac{h^{k}_{v}}{\left\| h^{k}_{v} \right\| }_{2}, \;\; \forall v \in V \end{aligned}$$
  where ${\left\| h^{k}_{v} \right\| }_{2}$ is the vector norm of the target node feature representation.

At the end of the forward propagation, the feature representations of the nodes are passed through a softmax layer which predicts the class labels for the nodes. The loss function computes the error using the predicted labels and actual labels. During the backward propagation, the gradient descent algorithm is used to optimize the weight matrix and the parameters in the aggregation function.

#### Boosting GraphSAGE

The DP-Net comprises of a boosting GraphSAGE model. The boosting GraphSAGE model uses multiple GraphSAGE models as the base estimators and the AdaBoost [29] algorithm to perform semi-supervised node classification.

It comprises of two phases: Initialization phase and Iterative phase. During the initialization phase the original dataset *X* is split into a train set $X_{train}$, test set $X_{test}$ and a validation set $X_{val}$ in the ratio of 70:15:15. Initially, the weight ($w_{i}$) of a sample ($s_{i}$) in the train set is assigned as $\frac{1}{N}$, where $i = 1, 2, \cdots , N$ and *N* is the total number of samples in the train set. The iterative phase comprises of M boosting rounds. At each boosting round, bootstrap samples of size N are drawn from the train set. These samples are used to train the base GraphSAGE at that round. Once the base GraphSAGE at a boosting round is learned, the train set is supplied as input to the model, which predicts the class labels of all the samples in the train set. The predictions are used to calculate the error rate and the importance of the base GraphSAGE. It should be noted that if the error rate is high then, the importance of the base GraphSAGE is low and vice versa. The importance of the base GraphSAGE is then used to update the weights of the samples in the train set for the next boosting round. Thus, the boosting GraphSAGE model works by assigning adaptive weights to each sample in the train set (i.e) the weights of the samples are changed at each boosting round. The sample weights also play an essential role in drawing the bootstrap samples (i.e.) the higher the weight of a sample, the higher the probability of the sample being included in the bootstrap. Formally, the steps in boosting GraphSAGE algorithm can be explained as follows

#### Initialization phase

- The entire dataset is split into train and test set, where the train set is used to train the M base GraphSAGEs in the boosting GraphSAGE model sequentially.
- The weight vector ${\varvec{w}}$ is initialized as $w_{i}=\frac{1}{N} \; \forall i = 1, 2, \cdots , N$

#### Iterative phase

For each base GraphSAGE $m\in \left\{ 1,2,\cdot \cdot M\right\}$

- Draw bootstrap sample $X_m$ of size *N* from $X_{train}$.
- Train the base GraphSAGE using $X_m$.
- Predict the class labels of all the samples in $X_{train}$ using the base GraphSAGE.
- Calculate the error $\epsilon$. The error is the sum of the weights of each of the misclassified samples in $X_{train}$ by the current base GraphSAGE. The error of the base GraphSAGE $\epsilon _m$ is computed using Eq. 11.
  11 $$\begin{aligned} \epsilon _{m}=\frac{\sum _{i=1}^{N}w_{i}^{m}{\mathbb {I}}(.)}{\sum _{i=1}^{N} w_{i}^{m}} \end{aligned}$$
  where the $w^{m}_{i}$ is the weight of sample $s_i$ at iteration *m*, ${\mathbb {I}}(.)$ is the indicator function which returns 1 if the sample $s_{i}$ is misclassified; otherwise it returns 0.
- If $\epsilon _{m}$ exceeds 0.5, discard the current base GraphSAGE and go to step (a) of Iterative phase.
- Calculate the weight $\alpha _{m}$ of the base GraphSAGE according to Eq. 12.
  12 $$\begin{aligned} \alpha _{m}=\frac{1}{2}ln\frac{1-\epsilon _{m}}{\epsilon _{m}} \end{aligned}$$
- Update the weights of the samples in $X_{train}$ using Eqs. 13 and 14
  13 $$\begin{aligned} w^{m+1}_{i}=w^{m}_{i}\cdot e^{ \alpha _{m}}, \;\; \forall i = 1, 2, \cdots , N \end{aligned}$$
  14 $$\begin{aligned} w^{m+1}_{i}=w^{m}_{i}\cdot e^{ -\alpha _{m}}, \;\; \forall i = 1, 2, \cdots , N \end{aligned}$$
- Normalize the weights of all the samples.

**Fig. 3:**
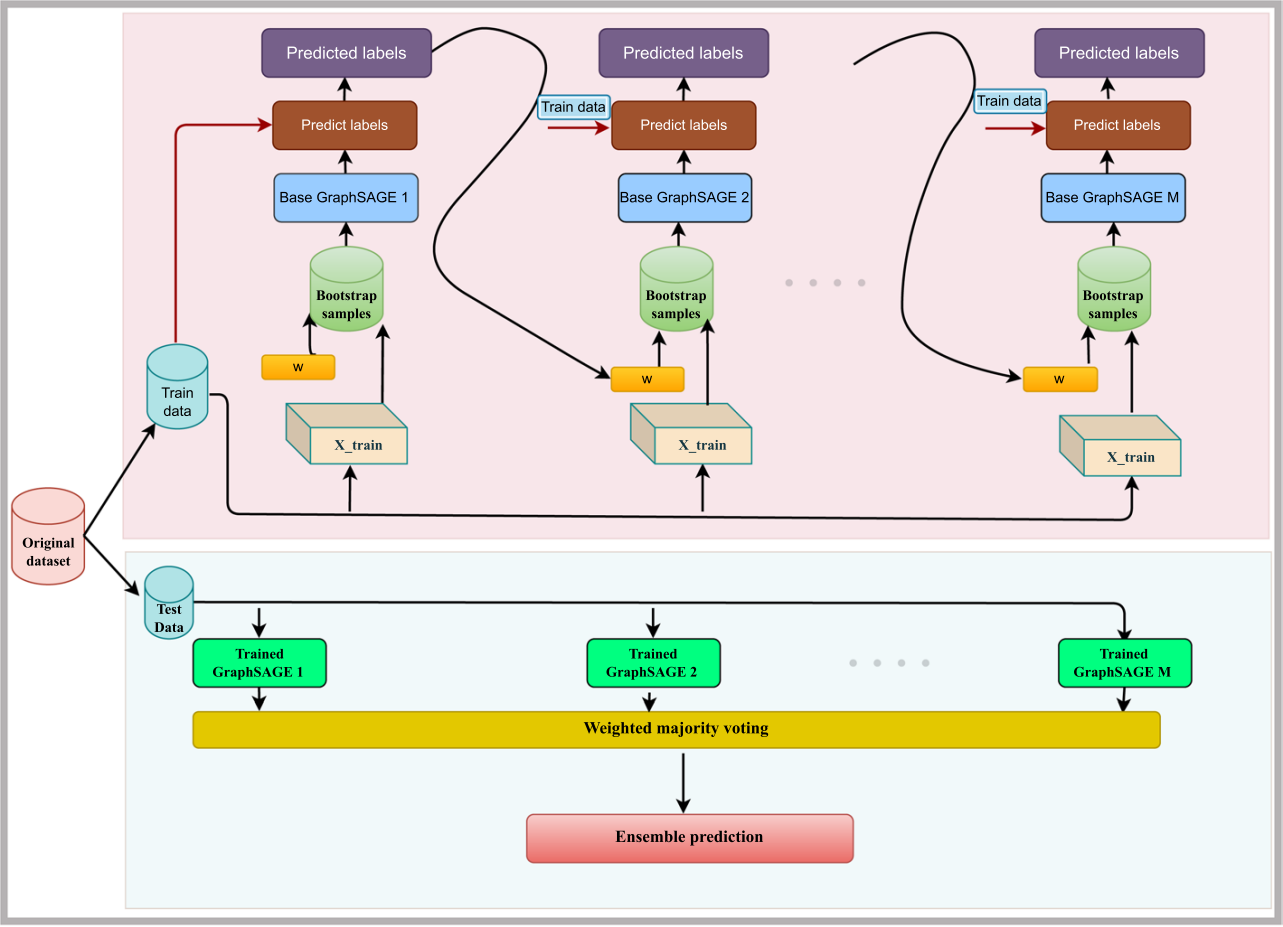
Architecture of boosting GraphSAGE: The top part of the figure illustrates the training phase where the base GraphSAGEs are learned. The bottom part of the figure illustrates the testing phase where the predictions of the individual trained base GraphSAGEs are combined together using weighted majority voting to produce the final prediction by the ensemble

The trained boosting GraphSAGE can be used to predict the category of a test sample ($x_{test}$). The prediction depends on the weighted majority voting and is given by Eq. 15.

15 $$\begin{aligned} H(x_{test})=sign\left( \sum _{m=1}^{M} \alpha _{m}h_{m}(x_{test})\right) \end{aligned}$$

where *H* is the final strong boosting GraphSAGE classifier, $H(x_{test})$ is the prediction made by *H* for the test sample, $h_m$ is the trained base GraphSAGE model at boosting round *m* and $h_m(x_{test})$ is the prediction made by $h_m$ for the test sample. According to the Eq. 15 the prediction of every base GraphSAGE weighted by its importance is summed up. The sign of the sum determines the final class label of the test sample. Figure 3 shows the schematic representation of boosting GraphSAGE

## Experimental setup

This section presents the implementation details, the experiments conducted, the parameter settings and the evaluation metrics.

### Implementation details

The proposed framework is written in Python3 programming language using Anaconda-Spyder environment. The libraries used in this work are Keras in TensorFlow version 2.9.2, Sklearn version 1.0.2, Pytorch version 1.13. The code is executed on an Ubuntu machine with 16 GB RAM, Intel i7 processor and a dedicated 4 GB NVIDIA GEFORCE.

### Experiments

The proposed boosting GraphSAGE model is learned using semi-supervised training. During training, the node representations for the train nodes are generated by the model. Also, the parameters and the aggregator functions are learned by the model. The model validation is done with the validation data. At the time of testing, the model uses the learned parameters and aggregator functions for generating the embeddings for test nodes. These embeddings are used by the model to predict the test node labels. In order to comprehensively investigate the effectiveness of the proposed model, the following experiments are conducted on all the datasets:

- Comparison of the proposed model with other ML, DL and ensemble models. The models used for comparison are as follows:
  ML models: SVM [30], MLP [31]
  Ensemble ML models: RF [32], DF [33], XGB [34]
  GNN models: single GCN [13], single GraphSAGE
  Ensemble GNN models: Bagging GCN, Boosting GCN, Bagging GraphSAGE, Boosting GraphSAGE
  Boosting GCN uses multiple base GCN models and the same adaptive boosting technique used by the boosting GraphSAGE model to train base GCN models. Bagging GCN and Bagging GraphSAGE use the bagging technique [35] to ensemble base GCN and GraphSAGE models respectively.
- Comparison of the proposed framework with previous work done on the datasets.

### Parameter settings

This section explains how the values of certain parameters of the various models experimented in this work are fixed.

#### Identifying the optimum number of neighbors and the best dissimilarity measure for each dataset

The best dissimilarity measure for constructing the edges between the nodes in the MD-graph is selected by conducting experiments using one base GraphSAGE model. The optimum number of neighbors for a node for a dataset is based on some percentage of the total number of samples in the dataset. In order to find the optimum number of neighbors for each dataset, experiments are conducted by varying the percentage value (Perc$\_$val) as 5, 10, 15, 20 and 25. Three dissimilarity measures are explored to find the best measure to compute the distance matrix for each dataset. A dissimilarity measure computes the dissimilarity between two graph nodes $v_i$ and $v_j$. Let ${\varvec{f}}_i \in {\mathbb {R}}^{1 \times f}$ be the feature vector of $v_i$ and ${\varvec{f}}_j \in {\mathbb {R}}^{1 \times f}$ be the feature vector of $v_j$. The dissimilarity measures between two graph nodes described by its features are defined as follows:

- Euclidean distance: $$\text {ED}(v_i, v_j)=\sqrt{\sum _{k=1}^{f}(f_{ik}-f_{jk})^2}$$.
- Manhattan distance: $$\text {ManDist}(v_i, v_j)=\sum _{k=1}^{f}\left| f_{ik}-f_{jk} \right|$$.
- Cosine dissimilarity: $1-\text {CS}(v_i,v_j)$ where $\text {CS}(v_i, v_j) =\frac{f_i \cdot f_j}{\left\| f_i \right\| \left\| f_j \right\| }$.

**Fig. 4:**
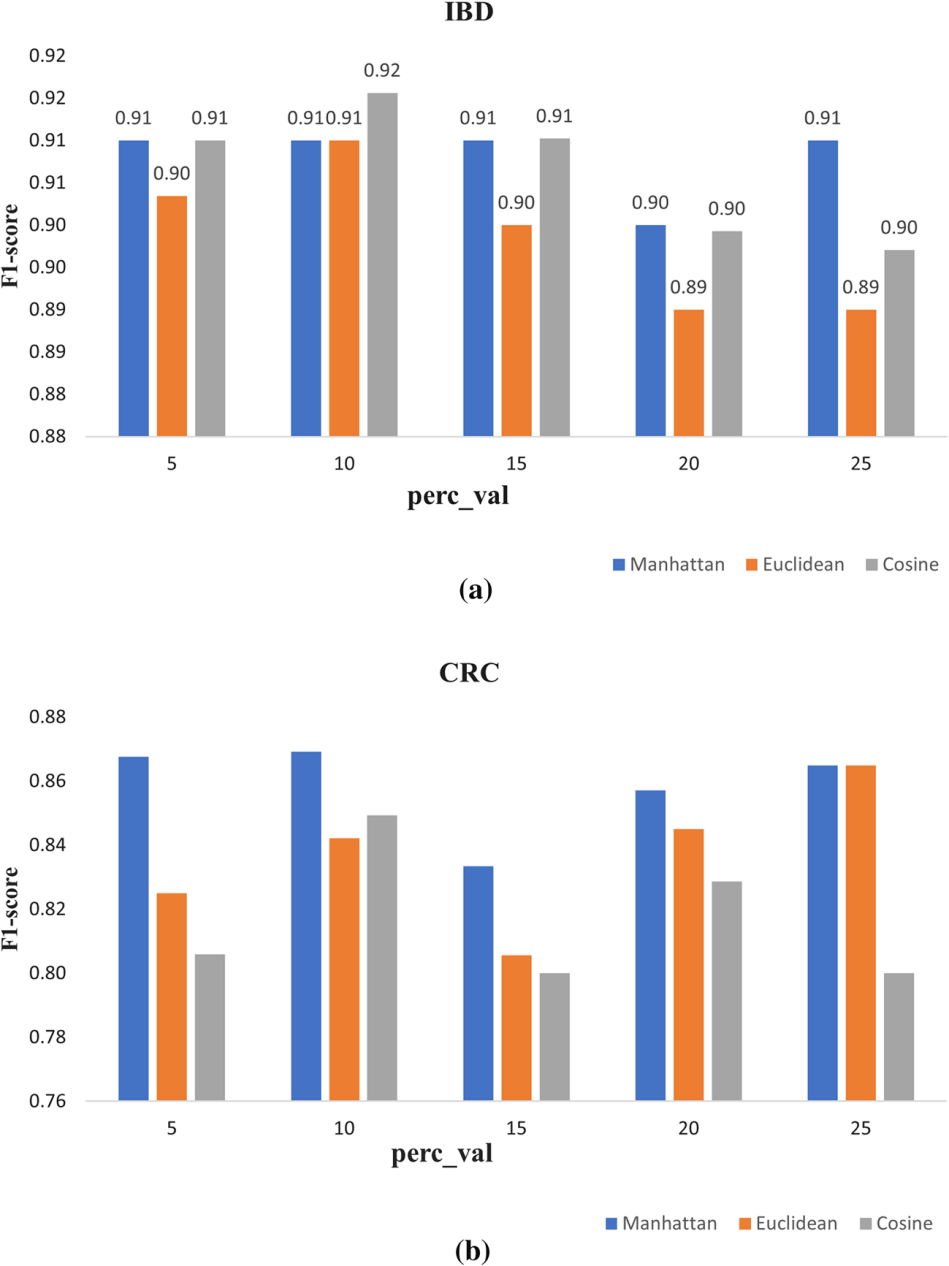
F1-score values achieved by single GraphSAGE for different dissimilarity measures for different $perc\_val$ on both datasets. **a** IBD dataset and **b** CRC dataset

The best dissimilarity measure out of the three measures (Manhattan, Euclidean, and Cosine) is selected based on the F1-score on the two real datasets. Figure 4 shows the F1-scores achieved by the GraphSAGE model on both real datasets for different dissimilarity measures and different values of $perc\_val$ parameter. As shown in Fig. 4a, the best F1-score of 92% for the IBD dataset is achieved when Cosine dissimilarity is used and the MD-graph constructed by restricting the number of neighbors as 10% of the total number of nodes. From Fig. 4b, it is evident that the best F1-score of 87% for the CRC dataset is achieved when Manhattan distance is used and the number of neighbors in the MD-graph is 10% of the total number of nodes. Thus, for the rest of this study, the neighbor threshold is set as 10% for both the datasets. Also, the dissimilarity measure for the IBD and CRC datasets are set as Cosine and Manhattan respectively.

#### Identifying parameters for single GraphSAGE and single GCN models

An empirical study is conducted on real datasets to decide the values for some parameters for the single GraphSAGE and, the single GCN models. For the single GraphSAGE and the single GCN models, experiments are conducted to decide on the optimum number of convolution layers. Additionally, for the single GraphSAGE model experiments are conducted to decide on the sample number of the first graph convolution layer ($S_{1}$) and the sample number of second graph convolution layer ($S_{2}$). The optimum number of convolution layers for the single GraphSAGE and single GCN models are found by varying the number of convolution layers in [2, 3, 4, 5] and recording the F1-scores obtained. To fix the best sample number for each convolution layer, a single 2-layer GraphSAGE model is used by varying $S_{1}$ in [10, 25, 50, 75] and $S_{2}$ in [5, 10, 25, 50]. The F1-score for each experiment is recorded.

**Fig. 5:**
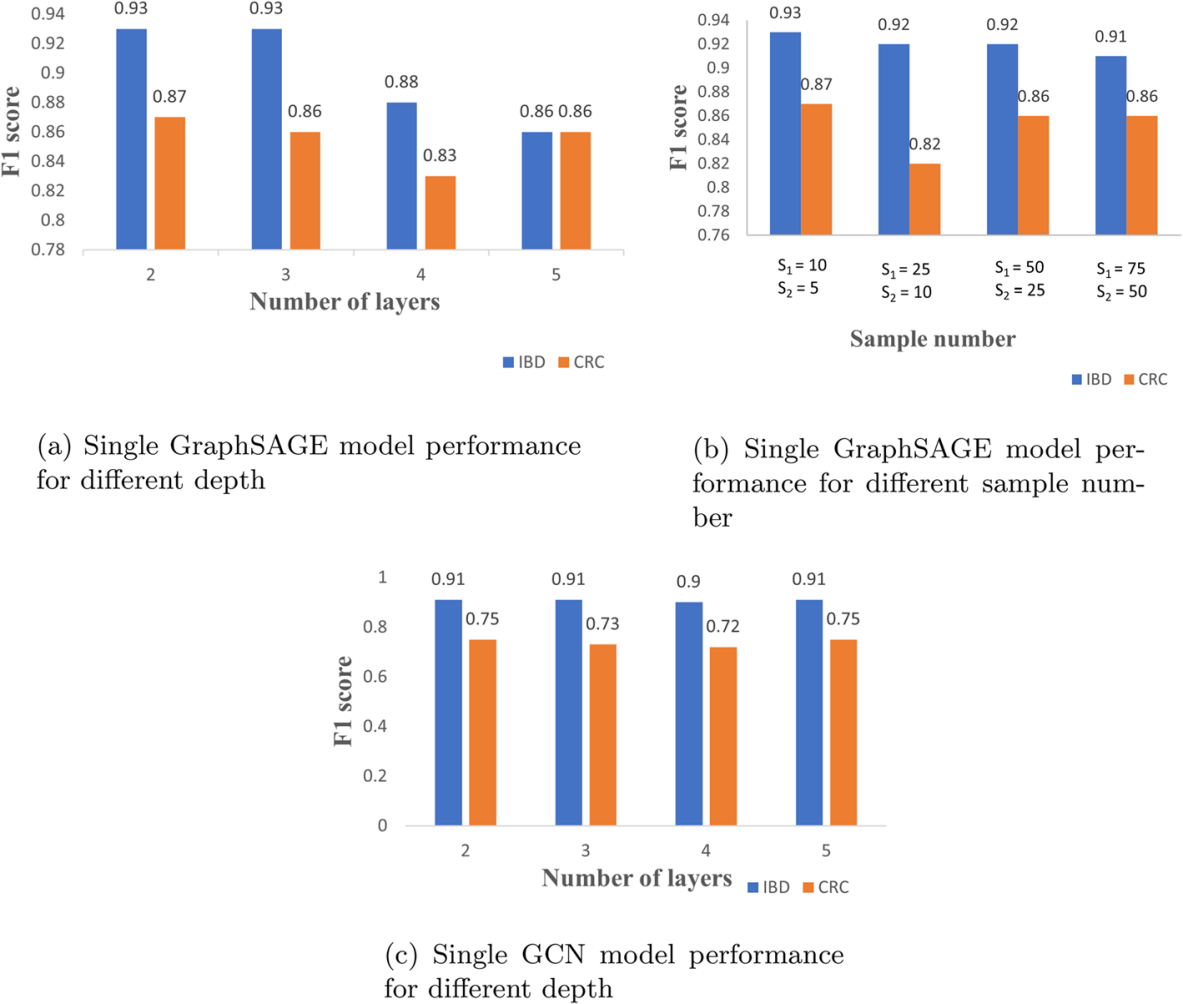
F1-score values achieved by GNN models

The bar plot in Fig. 5a shows that the single GraphSAGE model achieves the best F1-score of 93% on IBD and 87% on CRC datasets respectively, with two convolution layers. Hence, for further experiments with ensemble GraphSAGE, the number of layers is set as 2 for both datasets. The bar plot in Fig. 5b shows that the single GraphSAGE model achieves the best F1 score of 93% on IBD and 87% on CRC datasets respectively when $S_{1}$ equals to 10 and $S_{2}$ equals to 5. Hence, for further experiments with ensemble GraphSAGE, we set $S_{1}$ as 10 and $S_{2}$ as 5. Fig. 5c shows that the single GCN model achieves the best F1-score of 91% and 75% for IBD and CRC datasets respectively with two convolution layers. Hence, for further experiments on ensemble GCN, the number of layers of a single GCN model is fixed as 2. The values of other parameters of the graph models such as optimizer, learning rate, number of hidden units in each layer, activation function and loss function are fixed based on the analysis of these algorithms from literature [12, 18].

The parameters for the various GNN models are consolidated and presented in Table 3.

**Table 3:** Details of parameter settings for GNN models

Parameters	Bagging & boosting GraphSAGE	Bagging & boosting GCN
Number of convolution layers	2	2
Number of hidden units in each convolution layer	32	32
Sample number for layer 1	10	–
Sample number for layer 2	5	–
Activation function - convolution layer	ReLU	ReLU
Activation function - output layer	Softmax	Softmax
Epochs	50	50
Optimizer	Adam	Adam
Learning rate	5.00E−03	5.00E−03
Loss function	Categorical cross entropy	Negative log likelihood

#### Determining the optimum number of base classifiers for the ensemble graph models

The optimum number of base classifiers for the ensemble graph models is determined experimentally. For the ensemble GraphSAGE and GCN models, the number of base classifiers is fixed by conducting experiments by varying the number of base classifiers in [5, 10, 15, 20, 25, 30, 35,40] for both the real datasets. Then, the F1-score obtained from each experiment is recorded. The results are presented in Tables 4 and 5. From Table 4, it could be observed that for the IBD dataset, the best-performing boosting GraphSAGE model is obtained when the number of base classifiers is set to 10, the best Bagging GraphSAGE is obtained with 10 base classifiers, the best Boosting GCN is obtained with 30 base classifiers and the best bagging GCN is obtained with 15 base classifiers. For the CRC dataset, it could be observed from the Table 5 that the best boosting GraphSAGE is obtained with 15 base classifiers, the best Bagging GraphSAGE is obtained with 20 base classifiers, the best Boosting GCN is obtained with 30 base classifiers and the best bagging GCN is obtained with 25 base classifiers. Hence, in all experiments, the above mentioned values are used as the number of base classifiers for the ensemble graph models.

**Table 4:** F1-score of Ensemble GNN models for different number of base classifiers for the IBD dataset

Number of base classifiers	Boosting GraphSAGE	Bagging GraphSAGE	Boosting GCN	Bagging GCN
5	0.93	0.91	0.86	0.91
10	**0**.**95**	**0**.**94**	0.86	0.92
15	0.93	0.92	0.87	**0**.**94**
20	0.91	0.92	0.89	0.93
25	0.91	0.92	0.89	0.93
30	0.9	0.91	**0**.**92**	0.92
35	0.92	0.9	0.91	0.9
40	0.92	0.9	0.9	0.9

The bold font indicates highest results

**Table 5:** F1-score of Ensemble GNN models for different number of base classifiers for the CRC dataset

Number of base classifiers	Boosting GraphSAGE	Bagging GraphSAGE	Boosting GCN	Bagging GCN
5	0.82	0.83	0.76	0.75
10	0.83	0.85	0.76	0.81
15	**0**.**87**	0.85	0.81	0.82
20	0.85	**0**.**86**	0.82	0.8
25	0.8	0.86	0.8	**0**.**84**
30	0.85	0.85	**0**.**84**	0.82
35	0.85	0.83	0.82	0.81
40	0.83	0.82	0.81	0.81

The bold font indicates highest results

#### Parameters for ML, DL and ensemble models

All the ML models and ensemble ML models except RF are implemented using the default settings in their corresponding sklearn libraries. The RF model is constructed using the default settings from the random forest module in the sklearn library except for some parameters. The number of trees in this work is set to 200 and the criterion to to measure the quality of the a split is set to entropy.

### Evaluation metrics

To evaluate the performance of the proposed model, the widely used metrics for binary classification are used in this work. Let True Positive (TP) be the number of sick samples that are correctly predicted as samples with disease by a classifier model, False Positive (FP) be the healthy samples that are incorrectly predicted as samples with disease, True Negative (TN) be the healthy samples that are correctly predicted as healthy samples and False Negative (FN) be the sick samples that are incorrectly predicted as healthy samples.

Then, Accuracy (ACC) is the ratio of total number of predictions that are correct to the total number of predictions made and is given by the Eq. 16.

16 $$\begin{aligned} ACC = \frac{TP+TN}{TP+FP+TN+FN} \end{aligned}$$

Accuracy is not a good metric to use when the dataset has the class imbalance problem. So in this work F1-score is also used to evaluate the performance. It is the weighted harmonic mean of recall and precision and is given by the Eq. 17.

17 $$\begin{aligned} F1-score =\frac{2*TP}{2*TP+FN+FP} \end{aligned}$$

Area under operator curve (AUC) is the area underneath the receiver operator curve (ROC) that calculates the model’s ability to discriminate samples between positive and negative classes. The value of AUC ranges between 0 and 1. AUC value 0 indicates a poor classification and AUC value 1 indicates a perfect classification.

In order to evaluate the performance of the model when working with imbalanced datasets, a useful metric called area under the precision-recall curve (AUPRC) is also used. AUPRC is the area under the precision-recall curve, drawn with recall on the x-axis and precision on the y-axis. If the model is able to identify all of the positive examples (sick samples) without accidentally marking any healthy samples as sick, then the model achieves a perfect AUPRC. AUPRC value of 0 indicates a poor model and 1 indicates a perfect model.

## Results and discussion

### Comparison of the proposed model with other ML, DL and ensemble models

In this section, the capability of the boosting GraphSAGE method in classifying the metagenomic samples into sick and healthy class is compared with other ML, DL and ensemble models. The comparison is made according to the results achieved by each model in terms of the AUC, ACC, F1-score and AUPRC. Table 6 presents the summary of results achieved by all the models on real datasets. From the results it is evident that the boosing GraphSAGE model outperforms all the graph ensemble models, the single GraphSAGE, the single GCN, and other traditional ML and ensemble models in classifying the real disease datasets.

For the IBD dataset, the results show that ML models like SVM, RF, DF, XGB and MLP exhibit low F1-score. Among the ML models the SVM classifier achieves the best F1-score. One possible reason for the low performance of these classifiers can be the data imbalance. By analyzing the results of the single GNN models, the single GraphSAGE model outperforms the single GCN. In addition, the single GNNs outperform all the individual ML and ensemble ML models. Even though all the ensemble GNN models significantly improve the performance, the boosting GraphSAGE outperforms all the other models for all considered evaluation metrics. The boosting GraphSAGE achieves the best performance with an AUC of 93%, an ACC of 95%, an F1-score of 95% and an AUPRC of 95%.

For the CRC dataset, it is evident from the results that all the individual ML and ensemble ML models face difficulty achieving good results even though the dataset is balanced. The poor results may be due to the small sample size and high dimensionality of the dataset. From the results, it can be observed that SVM is the worst-performing classifier. The XGB performs better when compared to individual ML and ensemble classifiers. When analyzing the GNN models, it is observed that all the GNN models (i.e.) the single and ensemble models outperform the individual ML and ensemble models. Out of the GNN models, the single GCN performs poorly. The boosting and bagging GCN perform better than the single GCN but their performance is less than that of a single GraphSAGE model. However, the boosting GraphSAGE model achieves the best results, of 90% AUC, 91% ACC, 87% F1-score and 93% AUPRC.

Table 7 presents the summary of results achieved by all the models on synthetic datasets. The synthetic datasets have more samples than the real datasets and follow the same ratio of data imbalance as in the real dataset. As the number of samples increased in the synthetic dataset, the performance of all the models improved. From the results, it can be observed that for the IBD_synthetic dataset, the SVM classifier and all the variations of the GCN model (i.e., single GCN, boosting GCN, and bagging GCN) perform poorly when compared to other models with respect to ACC, AUC, F1-score and AUPRC. The RF classifier achieves good AUC but a reduced F1-score. Apart from SVM, RF and all the variations of the GCN models, other classifiers perform similarly on the IBD_synthetic dataset. The best AUC of 99% is achieved by RF, DF, MLP, XGB, and boosting GraphSAGE classifiers. The XGB classifier achieves the best ACC of 100%. However, DF, GraphSAGE, and boosting GraphSAGE classifiers also achieve a nearest ACC value of 99%. The best F1-score of 99% is achieved by boosting GraphSAGE, bagging GraphSAGE, and XGB classifiers. Similarly, the best AUPRC of 99% is achieved by DF, XGB, GraphSAGE, and boosting GraphSAGE classifiers.

The results of CRC_synthetic dataset show that the SVM, MLP and all the variations of the GCN model perform poorly when compared to the other models with respect to ACC, AUC, F1-score and AUPRC. The best AUC of 99% is achieved by boosting GraphSAGE, XGB, and RF classifiers. The boosting GraphSAGE achieves an ACC of 98%, which is slightly lower than the ACC achieved by GraphSAGE and the DF classifier. Also, the best AUPRC of 99% is achieved by RF classifier. However, the best F1-score is achieved by RF, DF, and all variations of GraphSAGE classifiers including the boosting GraphSAGE.

To sum up, the single GraphSAGE model is able to accurately discriminate the sick and healthy metagenomic samples in real and synthetic disease datasets. The sampling of neighbors and the aggregation of features from the sampled neighbors positively help the GraphSAGE model to get a better representation of the nodes and, thereby, effectively classify the nodes. Since, single GraphSAGE performs well, the ensemble GraphSAGE model improves the classification performance. Out of the two ensembles, bagging GraphSAGE and boosting GraphSAGE, the boosting GraphSAGE outperforms because the boosting GraphSAGE is able to tackle the misclassifications at each base classifier. However, it should be noted that for synthetic data, most of the ensemble ML models and the proposed model perform similarly. Comparing the results of ensemble ML models on real and synthetic datasets, the models achieve an increased performance in synthetic dataset because of the increased number of samples. But, still the proposed model is able to handle the data imbalance in both the real and the synthetic datasets. It could be observed from the results that the proposed model is not able to achieve the best ACC on both the synthetic datasets. However, it is evident that the performance of the proposed model exceeds all the other models for the real datasets.

**Table 6:** Summary of results on test set of real datasets

Dataset	Metrics	Classifiers										
		SVM	RF	DF	MLP	XGB	GCN	GraphSAGE	Boosting GCN	Bagging GCN	Bagging GraphSAGE	Boosting GraphSAGE
IBD	ACC	0.71	0.79	0.81	0.82	0.82	0.91	0.91	0.92	0.94	0.95	**0**.**95**
	F1-score	0.68	0.57	0.57	0.62	0.57	0.91	0.93	0.93	0.94	0.94	**0**.**95**
	AUC	0.70	0.80	0.80	0.81	0.83	0.88	0.89	0.89	0.91	0.92	**0**.**93**
	AUPRC	0.71	0.66	0.73	0.70	0.64	0.93	**0.95**	0.84	0.92	0.93	**0.95**
CRC	ACC	0.50	0.64	0.65	0.58	0.68	0.76	0.86	0.84	0.88	0.87	**0**.**91**
	F1-score	0.52	0.53	0.58	0.54	0.62	0.75	0.87	0.84	0.84	0.86	**0**.**87**
	AUC	0.53	0.67	0.67	0.57	0.73	0.76	0.87	0.85	0.87	0.87	**0**.**90**
	AUPRC	0.63	0.67	0.73	0.61	0.80	0.81	0.92	0.82	0.86	0.92	**0.93**

The bold font indicates highest results

**Table 7:** Summary of results on test set of synthetic datasets

Dataset	Metrics	Classifiers										
		SVM	RF	DF	MLP	XGB	GCN	GraphSAGE	Boosting GCN	Bagging GCN	Bagging GraphSAGE	Boosting GraphSAGE
IBD_synthetic	ACC	0.97	0.96	0.99	0.98	**1**.**00**	0.95	0.99	0.91	0.96	0.98	0.99
	F1-score	0.81	0.91	0.98	0.96	**0**.**99**	0.92	0.98	0.91	0.93	**0**.**99**	**0**.**99**
	AUC	0.92	**0**.**99**	**0**.**99**	**0**.**99**	**0**.**99**	0.95	0.98	0.90	0.95	0.98	**0**.**99**
	AUPRC	0.95	0.94	**0.99**	0.95	**0.99**	0.98	**0.99**	0.92	0.98	0.98	**0.99**
CRC_synthetic	ACC	0.87	0.98	**0**.**99**	0.92	0.97	0.89	**0**.**99**	0.87	0.89	0.98	0.98
	F1-score	0.75	**0**.**98**	**0**.**98**	0.91	0.96	0.88	**0**.**98**	0.93	0.87	**0**.**98**	**0**.**98**
	AUC	0.78	**0**.**99**	**0**.**99**	0.96	**0**.**99**	0.86	0.98	0.87	0.82	0.98	**0**.**99**
	AUPRC	0.82	0.99	0.98	0.94	0.96	0.92	0.98	0.94	0.92	0.98	0.98

The bold font indicates highest results

### Comparison with previous work done on the real datasets

To show the effectiveness of the proposed framework, a comparative analysis is conducted with the previous works [18, 27, 36] on the real datasets. Among these works, [18] and [36] have reported the results on the real IBD dataset and [27] has used the real CRC dataset. The metaNN model [18] used a 3-layer MLP to classify the samples of the real IBD dataset. The graph embedding deep feedforward network (GEDFN) [36] implemented feature selection using graph embedding and deep neural network to identify the best features for the real IBD dataset. [27] used a RF model for real CRC dataset classification. All these previous works have analyzed the results in terms of AUC value and hence this work compares the results in terms of AUC.

**Fig. 6:**
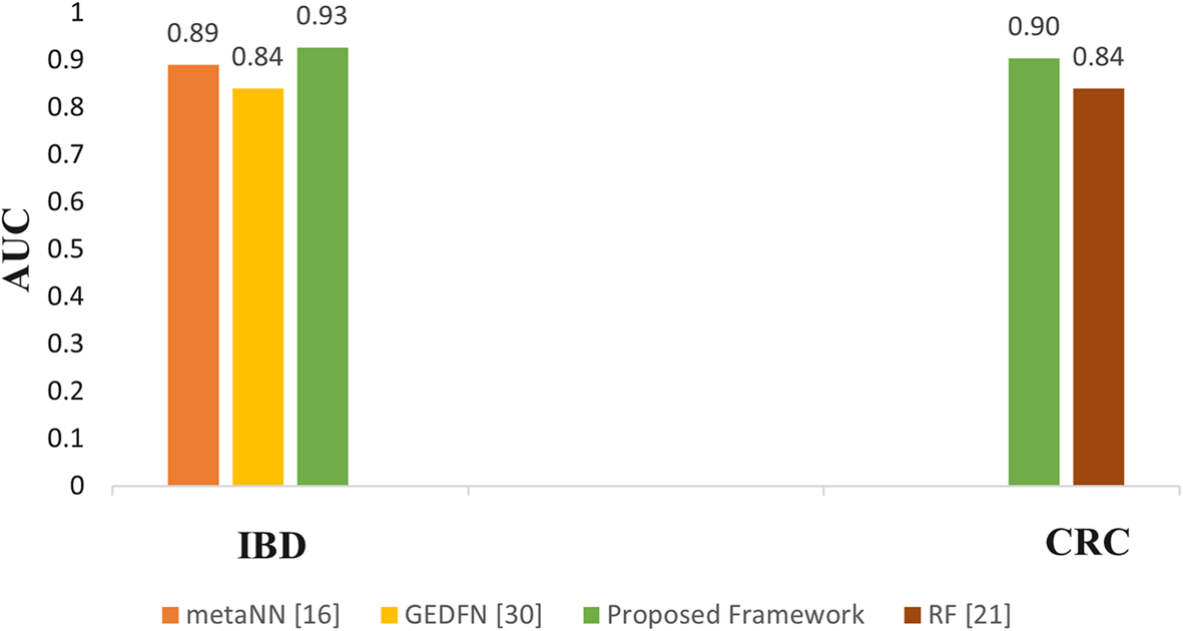
Comparison of AUC values with previous work done on the real IBD (left) and the real CRC datasets (right)

Figure 6 compares the AUC values achieved by the previous works and the proposed framework for the real IBD and the real CRC datasets. It can be seen from Fig. 6 that the proposed framework outperformed the previous works on both the datasets. The proposed framework achieves an AUC of 93% on the IBD dataset. However, the metaNN and GEDFN achieved an AUC of 89% and 84% respectively. Similarly, for the CRC dataset, the RF model obtained an AUC value of 84% while the proposed model achieves an AUC of 90%.

In summary, the improved results of the proposed framework prove that the boosting GraphSAGE model can effectively generate a powerful representation for each node by gaining relevant information from its neighbors. The ensembling technique has helped to improve the classification performance by overcoming the challenge of high dimensionality coupled with low sample size associated with metagenomic datasets. Additionally, the results also favor the modeling of the metagenomic datasets in the form of a graph for the application of graph based models to yield better performance. Furthermore, it should be noted that the proposed framework achieves considerable results for highly imbalanced datasets such as IBD, where all the other models failed to achieve good results. One reason for the improved result is the MD-graph construction procedure for the imbalanced dataset which has effectively addressed the imbalance problem by setting a different neighbor threshold for nodes from different classes. The different neighbor threshold helps the MD-graph to avoid unnecessary connections and to keep only the relevant relationship between the nodes.

## Case study

The case study aims to analyze the effectiveness of the MD-graph construction module of the proposed framework in capturing the neighbors of a graph node for the two real datasets.

Two sick samples S2, S10 and two healthy samples S7, S16 are considered from the real IBD dataset and the abundance of the microbial biomarkers in these samples is analyzed. The microbial biomarkers for the IBD disease are identified from few biological studies as Clostridiales, Enterobacteriaceae, Eubacterium, and Ruminococcus [37, 38]. While examining the adjacency matrix of the MD-graph, it is observed that the graph construction module is able to identify the sick samples (S2, S10) as neighbors and the healthy samples (S7, S16) as neighbors. This is because the sick samples exhibit high abundance values for the microbial biomarkers in their microbiome profiles. Also, the healthy samples exhibit low abundance values for the microbial biomarkers in their microbiome profiles. Table 8 shows the abundance values of the biomarkers in the microbiome profiles of samples S2, S10, S7 and S16 from the IBD dataset.

**Table 8:** Abundance of the microbial biomarkers in samples S2, S10, S7, and S16 of real IBD dataset

Biomarker	Sick samples		Healthy samples	
	S2	S10	S7	S16
Clostridiales	795	564	57	43
Enterobacteriaceae	5378	2150	41	26
Eubacterium	180	125	6	15
Ruminococcus	809	825	1	5

Similarly, two sick samples S7, S10 and two healthy samples S8, S11 are considered from the real CRC dataset and the abundance of the microbial biomarkers in these samples is analyzed. The microbial biomarkers for the CRC disease are identified from a few biological studies as *Peptostreptococcus, Streptococcus, Prevotella* and *Ruminococcus* [39]. While examining the adjacency matrix of the MD-graph, it is observed that the sick samples (S7, S10) are identified as neighbors and the healthy samples (S8, S11) are identified as neighbors in the MD-graph. This is because the sick samples exhibit high abundance values for the microbial biomarkers in their microbiome profiles. Also, the healthy samples exhibit low abundance values for the microbial biomarkers in their microbiome profiles. Table 9 shows the abundance values of the biomarkers in the microbiome profiles of samples S7, S10, S8, and S11 from the CRC dataset.

**Table 9:** Abundance of the microbial biomarkers in samples S7, S10, S8, and S11 of real CRC dataset

Biomarker	Sick samples		Healthy samples	
	S7	S10	S8	S11
Peptostreptococcus	933	715	8	3
Streptococcus	7161	6865	45	85
Prevotella	5258	3196	62	90
Ruminococcus	4554	3533	589	401

## Conclusion

In this paper, we have developed a new framework consisting of boosting GraphSAGE model to analyze human gut microbiome metagenomic data to classify the disease status as sick or healthy. In particular, the ability of GraphSAGE to work effectively on graph-based data is used for the binary classification task. We also proposed a novel graph construction approach using a dissimilarity measure for computing the dissimilarity between the samples. The graph was constructed by considering the data imbalance present in the dataset, which helped the proposed model to achieve the best results on the highly imbalanced dataset. The experimental results using real metagenomic disease datasets and synthetic datasets show that the proposed framework outperformed most competing methods in classification performance. Also, from the results, we confirm that the proposed framework can be effectively used for disease prediction and personalized medicine for microbiome-related diseases. In future, the proposed framework can be applied to other metagenomic and medical datasets for disease prediction.

## Data Availability

The real IBD and CRC datasets analyzed in this study can be downloaded from the MetaNN repository (https://github.com/ChiehLo/MetaNN) and SchlossLab’s repository (https://github.com/SchlossLab), respectively. The synthetic datasets generated/analyzed in this study can be downloaded from https://drive.google.com/drive/folders/15c59orR1aZAfC6rW_cXIaKaMLFI06Avl?usp=sharing.

## Abbreviations

ML: Machine learning
DL: Deep learning
OTU: Operational taxonomic units
CNN: Convolutional neural network
SVM: Support vector machines
RF: Random forest
DF: Deep forest
XGB: EXtreme gradient boosting
MLP: Multilayer perceptron
GNN: Graph neural network
GraphSAGE: Graph SAmple and aggreGatE
GCN: Graph convolutional network
IBD: Inflammatory bowel disease
CRC: Colorectal cancer
AUC: Area under the receiver operating characteristics curve
ROC: Receiver operating characteristics
T2D: Type 2 diabetes
NN: Neural network
NB: Negative binomial
