# Correction: Automatic disease prediction from human gut metagenomic data using boosting GraphSAGE

**DOI:** 10.1186/s12859-023-05431-9

**Published:** 2023-08-02

**Authors:** K. Syama, J. Angel Arul Jothi, Namita Khanna

**Affiliations:** 1 Department of Computer Science, Birla Institute of Technology and Science Pilani Dubai Campus, Dubai International Academic City, Dubai, UAE; 2 Department of Biotechnology, Birla Institute of Technology and Science Pilani Dubai Campus, Dubai International Academic City, Dubai, UAE

**Correction: BMC Bioinformatics (2023) 24:126** 10.1186/s12859-023-05251-x

Following the publication of the original article [1], the authors would like to correct affiliation 2.

The incorrect affiliation is: Nuclera Nucleics Ltd, Cambridge, UK

The correct affiliation is: Department of Biotechnology, Birla Institute of Technology and Science Pilani Dubai Campus, Dubai International Academic City, Dubai, UAE.

The original article [1] has been corrected.

## References

[1] Syama K (2023). Automatic disease prediction from human gut metagenomic data using boosting GraphSAGE. BMC Bioinform.

